# Glycine transport inhibitors for the treatment of chronic pain conditions: the time is ripe for clinical evaluation!

**DOI:** 10.3389/fnmol.2026.1793163

**Published:** 2026-03-17

**Authors:** Volker Eulenburg, Christopher L. Cioffi, Anutosh Roy, Robert J. Vandenberg

**Affiliations:** 1 Translational Anesthesiology and Intensive Care, Medical Faculty University of Augsburg, Augsburg, Germany; 2 Department of Chemistry and Chemical Biology, Rensselaer Polytechnic Institute, Troy, NY, United States; 3 School of Medical Sciences, University of Sydney, Sydney, NSW, Australia

**Keywords:** analgesia, chronic pain, glycinergic inhibition, GlyT1, GlyT2, neuropathic pain

## 1 Introduction

The treatment of chronic pain is—despite intensive research—still challenging, and the outcomes for patients are in many cases not satisfactory. Especially in patients suffering from neuropathic or nociplastic pain, the observed chronic symptoms are not only a manifestation of continuous nociceptive input but are the result of multiple maladaptive processes not only in the periphery, but also in the central nervous system, which complicates successful treatment. These changes result in a facilitated excitatory input from the periphery that is further aggravated at the level of the dorsal horn of the spinal cord by a reduction of inhibitory neurotransmission, in part, by changes of inhibitory synapse functions.

In line with these mechanistic explanations for the occurrence of chronic pain, the current gold standard for the treatment of chronic pain are the gabapentinoids, Gabapentin and Pregabalin that are thought to inhibit specific voltage gated Ca^2+^ channels (Guo et al., 2025) and thereby inhibit predominantly the excitatory input from peripheral C-fibers. Moreover, gabapentinoids are suggested to facilitate inhibitory neurotransmission by stimulating the descending noradrenergic system (Kremer et al., 2016). Similarly, the use of tricyclic antidepressants (TCAs), and serotonin–norepinephrine reuptake inhibitors (SNRIs) have been shown to ameliorate chronic pain by elevating serotonin and noradrenaline levels in the dorsal horn and by enhancing inhibitory neurotransmission in descending monoaminergic pain pathways, thereby indirectly enhancing inhibitory neurotransmission (Kremer et al., 2016). Although these treatment approaches have demonstrated efficacy in a subset of patients, a substantial proportion of patients derive little or no clinical benefit. In addition, both gabapentiniods and antidepressants can result in severe side effects that limit their acceptance by patients. Thus, in contrast to these rather indirect approaches, a direct approach, targeting inhibitory synapses directly to facilitate inhibitory neurotransmission in the dorsal horn appears promising. Here, the enhancement of GABAergic neurotransmission is the initial obvious choice since the large receptor heterogeneity suggests the possibility to identify receptor combinations exclusively expressed in the dorsal horn of spinal cord, thus allowing the facilitation of synaptic inhibition specifically in this brain region, without severe side effects. Despite promising results in mouse models, translation of these findings to clinical applications has failed due to side effects (Lara et al., 2020). Due to the very low glycine receptor (GlyR) heterogeneity, with only three alpha subunits in humans (four in mice) and one beta subunit, the facilitation of glycinergic neurotransmission also appears difficult.

A major focus is the enhancement of the activity GlyR α3, since this GlyR subunit is highly expressed in the superficial layers of the dorsal horn and it was demonstrated that phosphorylation-dependent downregulation of GlyR α3 receptor activity is involved in the development of inflammation-associated hyperalgesia and allodynia (Harvey et al., 2004). Indeed, pharmacological facilitation of GlyR α3 activity by a modified cannabinoid specifically binding to GlyR α3 was shown to ameliorate the facilitated pain response in animal models for both inflammatory and neuropathic pain (Lu et al., 2018). Similar effects were shown with substances enhancing the activity of both GlyR α3 and GlyR α1, thus demonstrating that stimulating glycinergic inhibition is a feasible approach. An alternative to the direct (allosteric) modulation of GlyR activity is the indirect facilitation of GlyR activity through inhibition of glycine transporters (GlyTs) GlyT1 and GlyT2, that have been shown to synergistically control extracellular glycine concentrations in the central nervous system (Cioffi and Guzzo, 2016). Based on the stoichiometry of ion-flux coupling of these transporters, GlyT1 will reduce synaptic concentrations to ~200 nM and GlyT2 will reduce it further to ~50 nM (Roux and Supplisson, 2000). Inhibition of each of these transporters has been shown to ameliorate the facilitated pain response in various animal models of chronic pain (Cioffi, 2018, 2021; Vandenberg et al., 2014).

## 2 GlyT1 inhibitors

Based on studies in GlyT1-deficient mouse models, as well as observations in human patients with GlyT1 encephalopathy, reduced GlyT1 activity is associated with a modest but measurable increase in extracellular glycine concentrations within the cerebrospinal fluid (CSF) (Kurolap et al., 2016). Consistent with these findings, pharmacological inhibition of GlyT1-mediated glycine uptake leads to a significant elevation of CSF glycine levels. This increase in extracellular glycine has been shown to enhance glycine receptor signaling (Gomeza et al., 2003) and to modulate NMDA receptor activity (Perry et al., 2008; Martina et al., 2004).

**Figure 1:**
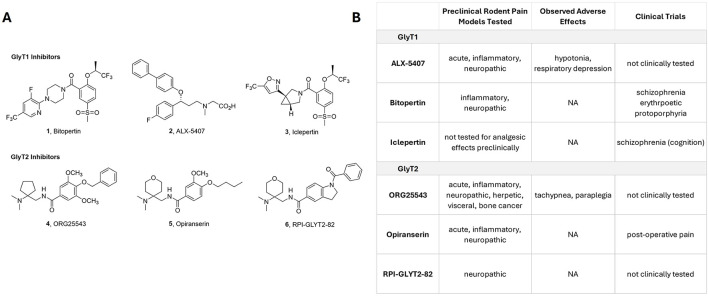
**(A)** Chemical structures of the GlyT1 inhibitors Bitopertin (1), ALX-5407 (2), Iclepertin (3), the GlyT2 inhibitors ORG25543 (4), Opiranserin (5), and RPI-GLYT2-82 (6). **(B)** Overview of the current stage of clinical development of the mentioned GlyT inhibitors, including animal tests performed in the context of acute and chronic pain, observed side effects.

Due to the broad expression of GlyT1 throughout the CNS, inhibition of this transporter is expected to elicit region-specific effects that depend on the local concentrations and physiological roles of glycine. Bitopertin (1, Figure 1) (Pinard et al., 2010), one of the most extensively studied GlyT1 inhibitors, was initially developed for the treatment of schizophrenia, with a particular emphasis on negative and cognitive symptoms. Although early clinical studies suggested potential benefit, subsequent Phase 2/3 trials produced mixed outcomes, and the program ultimately failed to meet its primary efficacy endpoint in a broad schizophrenia population (Bugarski-Kirola et al., 2014). Schizophrenia is associated, in part, with hypofunction of NMDA receptors, which are critical for synaptic plasticity, learning, and memory. By elevating extracellular glycine levels, GlyT1 inhibition was hypothesized to enhance NMDA receptor function and thereby improve cognitive deficits; however, this mechanistic rationale did not translate into consistent clinical benefit across patient populations.

More recently, bitopertin has been repurposed for evaluation in the rare hematologic disorder erythropoietic protoporphyria (EPP) (Ducamp et al., 2025), a condition characterized by painful photosensitivity and burning sensations. In this setting, bitopertin produced a significant reduction in the accumulation of the toxic metabolite protoporphyrin IX (PPIX), along with improvements in selected pain- and phototoxicity-related endpoints, although some measures did not reach statistical significance relative to placebo (Yeung et al., 2025). In preclinical studies, systemic administration of bitopertin increased mechanical and thermal withdrawal thresholds in rodent models of neuropathic pain (chronic constriction injury of the sciatic nerve) and inflammatory pain (carrageenan-induced paw inflammation) in a time- and dose-dependent manner (Armbruster et al., 2018). However, translation of these findings into human chronic pain indications has not yet been reported.

Other GlyT1 inhibitors, such as ALX-5407 (2, Figure 1) or Org25935, have also demonstrated robust analgesic effects in preclinical neuropathic pain models, accompanied by mechanistic evidence of modulation of the NMDA receptor NR1 subunit (Morita et al., 2008; Barthel et al., 2014). Both substances, most likely due to unfavorable pharmacodynamic properties that might results in severe side effects like respiratory despression, have not advanced into human pain trials, underscoring persistent translational challenges in this therapeutic area.

Interestingly, not only inhibitors, but also various GlyT1 substrates have been shown to be effective ameliorating facilitated pain response in preclinical animal pain models. This includes among others also N-ethylglycine (EG), a Lidocaine metabolite that was sufficient to reduce hyperalgesia and allodynia in animal models of chronic pain (Werdehausen et al., 2015) thus providing evidence, that at least part of the clinically observed general hypoalgesic effect of long term and/or systemic application of lidocaine might be mediated via GlyT1. A systematic analysis of the isolated effects of this lidocaine metabolite in humans, however, is still lacking.

## 3 GlyT2 inhibitors

GlyT2 inhibitors also have potential for elevating synaptic glycine concentrations to enhance glycinergic neurotransmission. Prolonged exposure to a selective GlyT2 inhibitor will elevate the resting glycine concentration from ~50 nM to ~200 nM. This will cause prolonged low level tonic stimulation of glycine receptors, although it might additionally limit the amount of glycine available for recycling of back into presynaptic vesicles for subsequent glycinergic neurotransmission. The first, and most well studied, GlyT2 inhibitor, ORG25543 (4, Figure 1) is a non-competitive inhibitor (Caulfield et al., 2001) and has analgesic properties in animal models for neuropathic pain (Dohi et al., 2009), but its effects in other pain conditions are controversial. ORG25543 is a high affinity, pseudo-irreversible inhibitor of GlyT2 (IC_50_ ~10 nM), and prolonged exposure of synapses to high concentrations of ORG25543 leads to a run-down in glycinergic neurotransmission due to depletion of pre-synaptic glycine concentrations. Whilst the analgesic effects of ORG25543 demonstrate the therapeutic potential of GlyT2 inhibitors, the side effects are a major concern. One approach to the side effect problem is to generate lower affinity inhibitors that transiently inhibit GlyT2 but also allow sufficient re-uptake to maintain availability of glycine for storage in synaptic vesicles.

One of these lower affinity GlyT2 inhibitors is opiranserin (5, Figure 1) which has a similar chemical scaffold as ORG25543 and shows analgesic efficacy (Pang et al., 2012). Opiranserin also has low affinity for serotonin transporters and serotonin type 2A receptors and it has been hypothesized that the activity at these additional sites also contributes to the analgesic activity. Opiranserin has undergone Phase 3 clinical trials for the treatment of acute post-surgical pain and has been demonstrated to reduce reliance on opioid-based drugs during the immediate post-surgical phase (Lee et al., 2024). Further investigations are required to establish whether opiranserin has potential for use in chronic or neuropathic pain conditions.

Recently, cryoEM structures of GlyT2 bound to ORG25543 and also a related compound, RPI-GLYT2-82 (6, Figure 1) have been determined (Cantwell Chater et al., 2026). RPI-GLYT2-82 is a lower affinity, non-competitive inhibitor that binds to a similar binding site to that of ORG25543. RPI-GLYT2-82 shows analgesic efficacy for neuropathic pain, without the side effects observed for ORG25543. However, it is also a substrate for P-glycoprotein, which may limit its blood brain barrier permeability, and reduce its therapeutic potential. Further refinement of this chemical scaffold has the potential to provide efficient blood brain barrier permeability and a new class of analgesics.

## 4 Perspective

Taken together, the currently available data suggest that, at least in animal models, inhibition of either GlyT1 or GlyT2 ameliorates the hypersensitivity and hyperalgesia observed in chronic pain conditions. Although the mechanism how these effects are elicited are not fully understood, a facilitation of glycinergic inhibition within the dorsal horn of the spinal cord is at least a major contributor to this effect, although additional effects, e.g., by facilitation of NMADR mediated glutamatergic input cannot be excluded. The analysis of the impact of GlyT1 inhibitors on the cerebrospinal fluid glycine concentration and its time course after application might provide information on the minimal effective dose for the treatment of chronic pain conditions and might provide additional information on the pharmacodynamic and pharmacokinetic properties of GlyT1 inhibitors. Correlation of these data might reveal optimization potential.

Despite these promising results, an application of GlyT inhibitors as monotherapy in clinical settings are, at least in the near future, unlikely. Thus, detailed analysis of co-application experiments, e.g., co-application of GlyT1 and GlyT2 inhibitors or combinations thereof with currently used substances like gabapentiniods, antidepressants, or substances facilitating GlyRs may provide important leads in the development of novel analgesic approaches. Here, potential additive effects might result in dosage reduction and thereby in a significant reduction of the observed side effects.

Finally, it must be considered, that despite the fact that animal models provide essential mechanistic information, they differ significantly in their pharmacodynamic and pharmacokinetic properties from humans. Initial human studies using the GlyT2 inhibitor, opiranserin, have already provided promising results for acute postoperative pain (Lee et al., 2025) with only moderate side effects observable only when very high dosages were used (Oh et al., 2018). Additional studies focusing on chronic pain where significant differences in the contribution of glycinergic neurotransmission on pain perception have been suggested, may provide important leads. The GlyT1 inhibitor Bitopertin, and also the recently published Iclepertin (Keefe et al., 2025) (3, Figure 1) have both been extensively tested in clinical trials for schizophrenia and a retrospective analysis of previous clinical data, as well as new studies, with a focus on chronic pain may prove important information in the optimal therapeutic use of GlyT1 inhibitors. Both substances have proven during large clinical trials to show sufficient bioavailabilty after oral uptake, be well tolerated and safe, while causing only mild adversary effects (Harvey et al., 2025; Bugarski-Kirola et al., 2016). The repurposing of GlyT1 inhibitors like Bitopertin for the treatment of rare hematological diseases like EPP, however, has stalled the evaluation in the context of chronic pain, that—in light of the relative high incidence in the population—would require large (and thereby expensive) clinical trails.

In summary, all available data corroborate the potential of GlyT inhibitors for the treatment of chronic pain. The major hurdle faced by researchers is the translation of promising preclinical studies to neuropathic pain conditions and identifying compounds that will provide optimal efficacy in humans. Future progress will depend on careful target selection, optimized inhibition profiles that preserve physiological glycinergic function, optimizing pharmacokinetic characteristics, and the identification of patient populations most likely to benefit from restored inhibitory signaling. As part of multimodal, opioid-sparing analgesic strategies, glycine transport inhibitors hold considerable promise to address the unmet need for effective and durable treatments for chronic pain conditions.
